# A Retrospective Study to Evaluate Discrepancies in Urban Versus Rural Mortality Due to Brain Cancer in the United States

**DOI:** 10.7759/cureus.60636

**Published:** 2024-05-19

**Authors:** Ram Kiran Maganti, Anubama Rajaravichandran, Vivek Kumar Patel, Sonika Suraparaju, Zuhaa Zahid

**Affiliations:** 1 Internal Medicine, Sri Devraj Urs Medical College, Kolar, IND; 2 Internal Medicine, PSG Institute of Medical Sciences and Research, Coimbatore, IND; 3 Internal Medicine, Gujarat Cancer Society Medical College, Ahmedabad, IND; 4 Internal Medicine, SVIMS-Sri Padmavathi Medical College for Women, Tirupati, IND; 5 Internal Medicine, Foundation University Medical College, Islamabad, PAK

**Keywords:** discrepancy, retrospective, cdc-wonder, urban, rural, brain cancer

## Abstract

Introduction: The study aims to examine the disparities in mortality rates attributed to brain cancer between urban and rural areas over a 22-year period, totaling 315,538 deaths. This investigation serves as a crucial step in identifying areas within healthcare that require improvement. By pinpointing the variations in mortality rates between urban and rural settings, healthcare authorities can strategically implement necessary interventions.

Methodology: A retrospective study was conducted by analyzing the death certificate available on the Centers for Disease Control and Prevention Wide-Ranging Online Data for Epidemiologic Research (CDC WONDER) database from 1999 to 2020 to evaluate the mortality rate trends of brain cancer ( International Classification of Diseases (ICD)-10 C71.0-71.9).The data was grouped based on rural and urban death rates according to the 2013 urbanization classification and the variables that were used were age, gender and race. Data was analyzed using Microsoft Excel and R Studio 4.3.1. Significant associations between demographic variables and mortality rates were identified via Binomial tests.

Results: From 1999 to 2020, urban areas recorded 259,402 deaths attributed to brain cancer, compared to 56,136 deaths in rural areas, indicating a higher mortality rate in urban settings. The mortality rate in both rural and urban areas exhibited an upward trend, except for a slight drop in 2010. The mortality rates were significantly higher in rural areas compared to urban areas for age groups 55-64 years and 65-74 years, males and caucasians.

Conclusions: Our research underscores the differences in death rates from brain cancer between urban and rural areas, specifically among individuals aged 55-64 and 65-74, males and those of caucasian ethnicity. Future research must adopt a multifaceted approach, integrating more recent datasets and embracing a finer granularity of individual-level information. Moreover, there is a pressing need to explore the interplay of various factors such as access to healthcare, treatment modalities, genetic predispositions, and socioeconomic determinants on mortality outcomes.

## Introduction

Brain cancer refers to the abnormal growth of cells in the brain, resulting in a variety of symptoms depending on the tumor type, size, and degree of spread. The term "brain cancer" encompasses both primary and secondary tumors involving brain tissue. Primary tumors include a wide range of malignancies originating from the CNS tissue, with glioblastomas accounting for 50.1% of them [1,2]. Primary malignancies of the brain have an annual incidence of around 7 per 100,000 persons, resulting in more than 15,000 deaths annually in the United States [3]. A nationwide population-based registry by Quinn et al. specifically focused on primary brain and other CNS tumors, providing statistical data including age and gender variability in incidence rates [4].

Patients from the most rural counties had a significantly higher risk of cancer death compared to those from urban counties [5]. Non-Hispanic (NH) caucasian individuals and males have the highest incidence of malignant brain tumors in the United States, while for non-malignant brain tumors, NH Black individuals and females have the highest incidence [6]. Among patients in the younger age group, NH caucasian individuals had a survival advantage compared to NH Black individuals, NH Asian or Pacific Island inhabitants, and Hispanics. In contrast, for the older age group (60-79 years), NH Black individuals, NH Asian or Pacific Island inhabitants, and Hispanics showed a survival advantage compared to NH caucasian individuals. This reflects the fact that survival disparities with respect to race and ethnicity disappear and sometimes even reverse for the older age group, primarily influenced by the poor survival rate among NH causasian individuals suffering from glioblastoma [7].

Rural residence has a varying effect on survival for patients with brain cancer, which can differ depending on the type of cancer. Previous studies have shown that incidence and mortality rates have a significant correlation with the human development index of the country, but the role of the degree of urbanization in affecting mortality-to-incidence ratios (MIRs) remains unclear. Exploring the disparities between urban and rural mortality rates is essential for establishing appropriate measures to address risk factors, reduce them, and customize treatment strategies accordingly [8].

Thus, the present study was undertaken in light of the paucity of research assessing discrepancies and trends between urban and rural mortality rates across different demographic variables for brain cancer.

Aims and objectives

The aim of this study is to assess the disparities in mortality in urban and rural areas for brain cancer using the Centers for Disease Control and Prevention Wide-Ranging Online Data for Epidemiologic Research (CDC WONDER) database, and identify any significant association between demographic variables and the mortality rates.

## Materials and methods

A retrospective original research study was conducted utilizing the CDC WONDER database, a comprehensive repository of public health data [9]. The data extraction process was carried out on March 27, 2024. As the study utilized deidentified, publicly available data from CDC WONDER, ethical approval from an ethics committee was not required.

CDC WONDER provides access to a wide range of health data, including mortality records dating back to 1999. Data collection for our study occurred on a single day from the CDC WONDER website, ensuring consistency and reliability of the dataset. The mortality analysis focused on the "1999-2020: Underlying Cause of Death by Bridged-Race Categories" category, allowing for comprehensive examination of mortality trends over a 22-year period.

To further refine our analysis, we selected the International Classification of Diseases (ICD) code for pulmonary embolism (I26) as the primary focus of investigation. Additionally, we categorized the data based on the Metropolitan 2013 classification, which divides geographical regions into Urban Metropolitan (including Large Central Metropolitan, Large Fringe Metropolitan, Medium Metropolitan, Small Metropolitan) and Rural/Non-metropolitan (including Micropolitan, Non-core) categories [10]. This classification was utilized in order to provide a nuanced understanding of mortality patterns across different urbanization levels.

Demographic factors included groupings by age (10-year age range), gender (male or female), and race (caucasian, Black/African-American, Asian/Pacific Islander and Alaskan Native/American Indian). The data was meticulously organized and exported to a Microsoft Excel sheet for further processing and analysis.

R Studio 4.3.1. was used for data and statistical analysis, with binomial tests employed to identify significant trends and associations within the dataset. p<0.05 was considered to be statistically significant.

## Results

Table 1 presents the absolute number of reported mortalities in urban and rural areas due to brain cancer from 1999 to 2020 according to the 2013 urbanization classification. The total number of deaths in the urban area, which includes Large Central Metropolitan, Large Fringe Metropolitan, Medium Metropolitan, and Small Metropolitan areas, is 259,402 over the 22-year period from the CDC WONDER site. The highest number of deaths was observed in the Large Central Metropolitan area (n=81,943; 31.6%), while the minimum number of deaths occurred in the Small Metropolitan area (n=31,899; 12.3%). The total number of deaths in rural areas, including Micropolitan and Non-core areas, was found to be 56,136. Among rural areas, the highest number of deaths occurs in the Micropolitan area (n=31,861; 56.8%), while the minimum number of deaths occurs in the Non-core area (n=24,275; 43.2%).

**Table 1:** Absolute number of reported mortalities in urban and rural areas due to brain cancer from 1999 to 2020 as per 2013 urbanization classification

Type	N (%)
Urban (Metropolitan area)	2,59,402
Large Central Metropolitan	81,943 (31.6%)
Large Fringe Metropolitan	77,841 (30%)
Medium Metropolitan	67,719 (26.1%)
Small Metropolitan	31,899 (12.3%)
Rural (Non-metropolitan Area)	56,136
Micropolitan	31,861 (56.8%)
Non-core	24,275 (43.2%)

**Figure 1:**
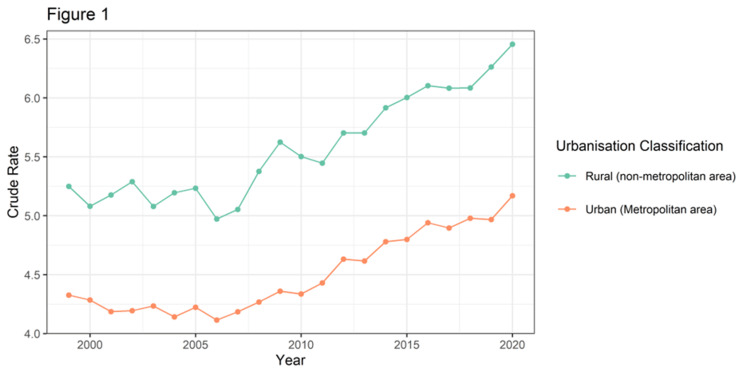
Line diagram showing trends in urban versus rural mortality due to brain cancer calculated in crude rate per 100,000 population

Figure 1 displays the trends in urban versus rural mortality due to brain cancer along with the calculated crude rate per 100,000 population. The mortality rate in rural areas has consistently been higher than in urban areas for brain cancer over the past 22 years, from 1999 to 2020. The crude death rate in rural areas is 5.3 versus 4.3 in urban areas for the year 1999. The crude death rate in rural versus urban areas for the year 2020 is 6.4 versus 5.2. The mortality trend in urban areas shows a rising/decreasing pattern until the year 2006, followed by a sharp increase in mortality until 2009, then it follows a rising/decreasing pattern until 2020. The mortality trends in rural areas also show a rising/decreasing pattern until 2006, followed by a rise until 2009, then again following a rising/decreasing pattern until 2020. Throughout these 22 years, the mortality rate is higher in rural areas for brain cancer compared to urban areas.

Table 2 presents mortality due to brain cancer based on age, gender, and race. Based on the 10-year age group, mortality is higher in people above 85 years of age and lower in children less than one year of age. There is no statistically significant difference in the number of deaths between urban and rural populations for age groups less than 1 year, 1-4 years, 5-14 years, 75-84 years, and more than 84 years, with a p-value of more than 0.005. For all other age groups, the number of deaths in rural areas is higher than in urban areas, with a statistically significant p-value of less than 0.001.

**Table 2:** Mortality due to brain cancer in urban and rural area based on age, gender and race *p value < 0.05; statistically significant

Variables	Urban		Mortality Rate (per 100,000)	Rural		Mortality Rate (per 100,000)	Binomial Test
	Mortality	Total population		Mortality	Total population		P value
Age Groups							
< 1 year	258	74,818,291	0.345	44	12,095,361	0.364	0.698
1-4 years	1,758	299,561,638	0.59	282	49,008,275	0.58	0.768
5-14 years	5841	770,126,283	0.76	975	131,095,742	0.74	0.557
15-24 years	4,051	799,755,615	0.51	800	136,038,105	0.588	<0.001*
25-34 years	7,193	803,054,375	0.9	1,418	117,033,859	1.22	<0.001*
35-44 years	16,084	803,916,304	2.0	3,120	127369780	2.45	<0.001*
45-54 years	34,952	788,609,639	4.43	6,962	138,965,498	5.0	<0.001*
55-64 years	58,772	639,136,856	9.2	12,738	127,286,485	10.0	<0.001*
65-74 years	63,533	417,988,177	15.2	15,031	92,469,123	16.26	<0.001*
75-84 years	48,735	244,183,949	19.96	11,007	54,320,144	20.26	0.112
85+ years	18,221	98,324,522	18.5	3,758	21,189,280	17.75	0.007*
Gender							
Male	145,230	2,815,055,490	5.159045728	31,611	502,292,400	6.293	<0.001*
Female	114,172	2,924,420,159	3.904090171	24,525	504,579,252	4.86	<0.001*
Race							
American Indian or Alaska Native	681	62,750,775	1.085245561	514	25,610,416	2.0	<0.001*
Asian or Pacific Islander	6,486	359,973,366	1.80179997	177	11,940,089	1.48	0.009*
Black or African-American	17,709	830,766,851	2.131644995	2210	88,267,994	2.50	<0.001*
Caucasian	234,526	4,485,984,657	5.227971514	53235	881,053,153	6.04	<0.001*

Based on gender, mortality due to brain cancer is higher in males than in females in both urban and rural areas. Mortality due to brain cancer is higher in rural areas for males compared to urban areas, with a statistically significant p-value of less than 0.001. For females, mortality is higher in rural areas than in urban areas, with a p-value of less than 0.001.

Based on race, the number of deaths is lower in the American Indian or Alaska Native population compared to other races in both urban and rural areas. The number of deaths due to brain cancer is higher in the caucasians in both urban and rural areas. There is no statistically significant difference in mortality between urban and rural areas for the Asian or Pacific Islander population, with a p-value of 0.009. For all other races, including American Indian or Alaska Native, Black or African-American, and caucasian, mortality is higher in rural areas than in urban areas, with a statistically significant p-value of less than 0.001.

## Discussion

The mortality rate in rural areas has consistently been higher than in urban areas for brain cancer over the past 22 years, from 1999 to 2020. Based on demographic characteristics, the crude mortality rates were observed to be higher in people above 85 years of age, males, and in the caucasians in both urban and rural areas. One possible explanation for the higher rates of mortality could be the improved accuracy and sensitivity of diagnostic tests, leading to the identification of cases that may have been previously "missed" or undiagnosed [11].

Our research reveals a notable shift in malignant brain tumor mortality rates over the years, characterized by a decline from 1999 to 2006 followed by an upward trend from 2006 to 2020. The initial decrease in mortality aligns with findings from Gittleman et al., who reported a consistent annual decrease of 0.9% in brain tumor mortality from 1991 to 2012 [11]. Conversely, the subsequent rise in mortality rates is corroborated by Miller et al., whose study indicates a yearly increase of 0.4% in mortality rates from 2009 to 2018 [12].

Furthermore, our analysis highlights a higher age-adjusted mortality rate (AAMR) in males, a trend consistent with the research of Ostrom and Thierheimer et al. [13,14]. This observation is likely linked to the elevated incidence of malignant brain tumors in males, particularly in cases of glioblastoma, which are reported to be 1.6 times more prevalent in males than in females. Sex-related disparities in genetic factors may contribute to the observed mortality trends. Sun et al. elucidated that at the molecular level, male glioblastoma cells exhibit a more pronounced inactivation of the tumor suppressor protein RB compared to their female counterparts [15]. This novel insight suggests that male astrocytes may inherently possess a greater susceptibility to malignant transformation, potentially impacting treatment response.

Moreover, the highest mortality rates were observed among NH caucasian individuals, while the lowest rates were seen in Asian or Pacific Islanders. One plausible explanation lies in the racial distribution of tumor histopathology. Studies indicate a higher proportion of high-grade gliomas (such as glioblastoma, astrocytomas, and nerve sheath tumors) in caucasian individuals, while Black individuals tend to have a higher proportion of lower-grade tumors [12,16]. These disparities may stem from variations in tumorigenesis among different racial groups [17].

Presently, there exists no research directly comparing mortality rates for brain tumors between rural and urban areas. However, numerous studies have illustrated a connection between socioeconomic status (SES) and survival rates [18, 19]. Cote et al. unveiled that disparities in survival were attributable to SES rather than the rural-urban divide [18]. This contrast may be attributed to an individual's insurance status, which serves as a proxy for SES. Individuals with higher SES often have the means to afford private insurance, granting them better access to quality medical facilities and advanced treatment options, such as novel chemotherapies in clinical trials and radiotherapy [19]. Conversely, those with Medicaid or lacking insurance typically belong to lower SES groups, facing limited access to screening tools and being less likely to undergo surgical resection or biopsy. Consequently, they may present late with a larger tumor burden [19].

Research also shows that rural populations often face higher mortality rates and poorer health outcomes due to limited access to healthcare. For instance, a study by Baljepally and Wilson found higher cardiovascular disease rates in rural areas, while another by Borkowski and Borkowska highlighted challenges in HIV treatment access for rural residents [20,21]. These examples underscore the need for targeted interventions to address urban-rural health disparities across various diseases.

Limitations

The most recent data from 2021 to 2023 has not been incorporated into the analysis. The study lacks individual-level data, such as comorbidity burden, disease duration, histopathological types, medical treatments, or prior interventions, all of which can impact mortality rates and serve as confounding factors.

Furthermore, brain cancer was not categorized based on sub-categories, and causes of death specific to the disease were not investigated as the data was sourced from CDC WONDER, which does not provide such detailed information. Similarly, socioeconomic factors and access to healthcare could not be examined.

## Conclusions

In conclusion, our retrospective study utilizing CDC WONDER data revealed a concerning trend of increasing mortality rates in rural areas over the past 22 years, particularly evident in the 85+ age group, males, and caucasian individuals. While SES appears to play a crucial role in survival outcomes, the lack of individual-level data and categorization by subtypes limits our understanding of the underlying factors contributing to these disparities.

Future research must adopt a multifaceted approach, integrating more recent datasets and embracing a finer granularity of individual-level information. Moreover, there is a pressing need to explore the interplay of various factors such as access to healthcare, treatment modalities, genetic predispositions, and socioeconomic determinants on mortality outcomes. It is imperative for a deeper understanding of brain cancer mortality trends and the development of targeted interventions to address disparities.
